# Evidence for the existence of powder sub-populations in micronized materials: Aerodynamic size-fractions of aerosolized powders possess distinct physicochemical properties.

**DOI:** 10.1007/s11095-014-1414-3

**Published:** 2014-07-12

**Authors:** Sara Jaffari, Ben Forbes, Elizabeth Collins, Jiyi Khoo, Gary P Martin, Darragh Murnane

**Affiliations:** 1 Institute of Pharmaceutical Sciences, King’s College London, Stamford Street, London, SE1 9NH UK; 2 Pfizer Global R&D, Sandwich, Kent, UK; 3 Surface Measurements Systems Ltd, 5 Wharfside Rosemont Road, Middlesex, HA40 4PE UK; 4 Department of Pharmacy, Centre for Research in Topical Drug Delivery and Toxicology, University of Hertfordshire, College Lane, Hatfield, AL10 9AB UK

**Keywords:** dispersion, distribution, heterogeneity, inhaled drug delivery, intra-batch variability, surface energy

## Abstract

**Purpose:**

To investigate the agglomeration behaviour of the fine (<5.0 μm) and coarse (>12.8 μm) particle fractions of salmeterol xinafoate (SX) and fluticasone propionate (FP) by isolating aerodynamic size fractions and characterising their physicochemical and re-dispersal properties.

**Methods:**

Aerodynamic fractionation was conducted using the Next Generation Impactor (NGI). Re-crystallized control particles, unfractionated and fractionated materials were characterized for particle size, morphology, crystallinity and surface energy. Re-dispersal of the particles was assessed using dry dispersion laser diffraction and NGI analysis.

**Results:**

Aerosolized SX and FP particles deposited in the NGI as agglomerates of consistent particle/agglomerate morphology. SX particles depositing on Stages 3 and 5 had higher total surface energy than unfractionated SX, with Stage 5 particles showing the greatest surface energy heterogeneity. FP fractions had comparable surface energy distributions and bulk crystallinity but differences in surface chemistry. SX fractions demonstrated higher bulk disorder than unfractionated and re-crystallized particles. Upon aerosolization, the fractions differed in their intrinsic emission and dispersion into a fine particle fraction (<5.0 μm).

**Conclusions:**

Micronized powders consisted of sub-populations of particles displaying distinct physicochemical and powder dispersal properties compared to the unfractionated bulk material. This may have implications for the efficiency of inhaled drug delivery.

## INTRODUCTION

Dry Powder Inhaler (DPI) formulations generally consist of micronized drug (<5.0 μm) in an agglomerated form which may be blended with large crystals of lactose monohydrate functioning as both a drug carrier and bulking agent. Frequently, a fine particle excipient such as micronized lactose is also incorporated into the formulation to help facilitate drug particle de-agglomeration [1]. Pharmaceutical powders, however, are complex non-homogenous systems; this renders the formulation of DPIs, such that high pulmonary delivery is achieved, a challenge. Powder properties can vary widely; for example, there is often a broad particle size distribution (PSD) [2, 3] and this alone is a predominant factor in determining the mechanism and location of drug deposition in the lungs [4]. The physicochemical properties of the particulate system, such as particle size [5–7], shape [8], surface roughness [9, 10], surface energy/interfacial chemistry [11–13] and crystallinity [3, 14] are therefore crucial to the aerodynamic behaviour of the DPI [2]. Such factors can influence the aerosolization, *in vitro* and *in vivo* deposition profiles, and bioavailability of the drug [1], and therefore have important consequences with regards to the efficacy of the inhaled treatment.

In addition to the inherent variability in powder properties, processing steps such as milling/micronization and blending can often also introduce an additional source of particle heterogeneity [15]. Micronization, although being a widely adopted method for size reduction, can be inefficient and is capable of inducing physical and chemical changes in particles that can affect the performance of the inhaled formulation [1, 15–17]. The process of micronization also offers limited opportunity to control and/or manipulate particle properties [18], such that inter-batch variability in surface energy [15, 19], powder flow [15], and cohesivity/agglomeration propensity [20] can arise impairing the quality, efficacy and performance of the final marketed product [15]. The blending protocol employed during formulation can further alter powder characteristics [21], and may result in changes to the fluidisation and/or aerosolization behaviour of the powder [21–23].

Another consequence of poorly controlled mechanical comminution which is less well characterised is intra-batch variability. Differing levels of processing stress experienced across various sites within a powder bulk can lead to micro-areas within the powder which possess different properties [24], and this may be manifest as altered flowability and aerosol dispersion/de-agglomeration behaviour [24, 25]. Mechanically micronized powders also exhibit a ‘dynamic nature’ in which thermodynamically activated amorphous sites on the surface of the particle can as a function of time revert back to crystalline material [18]. Therefore any changes in powder properties, such as surface energy and crystalline disorder, can further contribute towards the potential for intra-batch differences within powders.

Recently, the next generation impactor (NGI) has been used to isolate powder fractions preparatively based on their aerodynamic particle size [26]; this is achieved by dispersing powder into the impactor and recovering deposited material from the stages. By maintaining a constant flow rate through the NGI, the aerodynamic particle size of the deposits can be calculated. Operation of the impactor is not affected by the physicochemical properties of the powder, thus by characterising the fractions for their aerosol performance, for example, any differences can be attributed to particle physicochemical properties rather than aerodynamic size [26].

The aim of this study was to investigate the agglomeration behaviour of the fine (<5.0 μm) and coarse particle (>12.8 μm) fractions of salmeterol xinafoate (SX) and fluticasone propionate (FP) by isolating these aerodynamic size fractions from micronized bulk powders. The fractions were subjected to physicochemical characterisation to determine the particle size, morphology, crystallinity and surface energy distribution, and an assessment of aerosol performance was also made.

## MATERIALS AND METHODS

### Materials

Salmeterol xinafoate (SX) and fluticasone propionate (FP) were obtained from Vamsi Labs, India (BN. SX-0081010) and LGM Pharma, USA (BN. 5501-B-11030), respectively. Cyclohexane was purchased from VWR International Ltd, UK. Sorbitan monooleate 80 (Span 80) and polypropylene glycol 400 (PEG400) were from Sigma Aldrich Ltd, UK. Methanol (Fisher Scientific Ltd, UK) and ammonium acetate (Chromanorn Hipersolv for HPLC, BDH Prolabo, VWR International Ltd, UK) were high performance liquid chromatography (HPLC) grade. Whatman^TM^ nylon filters (pore size 0.2 μm and 0.45 μm, diameter 47 mm) and hexane were purchased from Fisher Scientific Ltd, UK. Polypropylene glycol (average Mn approx. 1000) was from Sigma Aldrich Ltd, UK, and size 3 gelatin capsules were from Capsugel, France.

## Methods

### Particle Preparation

#### Aerodynamic Fractionation

Aerodynamic fractionation was conducted as previously described [26]. Powder (1–2 g) was aerosolized into the NGI (MSP Corporation, USA, supplied by Copley Scientific, UK) at a flow rate of 60 L.min^−1^ using a Malvern QSpec dry powder feeder (DPF, Malvern Instruments Ltd., UK). The NGI was assembled as described in the British Pharmacopoeia 2012; solvent was not added to the pre-separator and the plates were used uncoated. The vacuum pump (Twin Impinger Model, TI2) and DPF were switched on for 2 min during which powder was aerosolized into the impactor. Following each 2 min interval, the NGI was dismantled, and the powder deposits were carefully recovered using a plastic scraper and transferred into clean, dry glass vials. This was repeated until a yield of approximately 1 g was obtained in the pre-separator and at least two of the fine particle stages for each powder.

#### Amphiphilic Crystallization

Amphiphilic Crystallization was conducted as previously described [27–30]. PEG400 solutions containing 4.5% w/w SX or 0.65% w/w FP were prepared and subjected to high shear mixing (Model L4RT laboratory homogeniser, Silverson Machines, USA) at 2,100 rpm for SX and according to the following protocol for FP: 2,000 rpm for 10 min, 3,000 rpm for 10 min, and 1,000 rpm for 5 min. Following dissolution, the solutions were degassed by ultrasonication for 5 min, and filtered (0.2 μm hydrophobic PTFE syringe filter, Whatman^TM^). The addition of anti-solvent (water) was in the ratio 1:11 (solution:water) for SX and 1:7 for FP, and occurred at a rate of 20 g.min^−1^.g^−1^ of solution whilst being stirred using an overhead stirrer (Model RZR 2051 2051, Heidolph,UK) at 1,000 rpm for SX solutions, and ramped from 700 to 1,430 rpm during the period of water addition for FP solutions. The crystals were harvested by vacuum filtration (0.2 μm nylon filter, Whatman^TM^) and dried overnight at 50°C in a vacuum oven. The dried powder was washed with 200 mL cold water (4°C) with the aid of stirring at 1,470 rpm for 5 min, harvested by filtration, and dried. The following day the crystals were de-caked by sonicating (5–6 min) with 15 mL cyclohexane and vacuum drying (50°C) for 3 h.

### Particle Characterisation

#### Particle Size Analysis

Laser diffraction particle sizing was carried out using a Malvern Mastersizer X (Malvern Instruments Ltd, UK) fitted with a 100 mm focal length lens (0.5–180 μm) and an MS7 magnetically stirred cell as described previously [31]. Drug-saturated solvent dispersants were prepared consisting of Span 80 (0.5% w/v for SX, 0.1% w/v for FP) in cyclohexane. Dispersants were sonicated for 30 min followed by overnight stirring. Approximately 1 mg of drug was added to 2 mL filtered dispersant (0.2 μm cellulose acetate syringe filter) and sonicated for 5 and 2.5 min for SX and FP respectively (Sonicleaner, DAWE, Ultrasonics Ltd, USA). A background reading was taken and the suspension was added to the sample cell until the obscuration was ~10–30%. Following equilibration (60 s), ten individual measurements (measurement sweeps 2500 for SX and 3500 for FP) were taken for *n* = 4–6 samples to obtain particle size measurements calculated using Mie theory. Particle size metrics considered were the D_v10_, D_v50_, and D_v90_, corresponding to the particle size below which 10%, 50% and 90% of the particles, by volume, are smaller, in the distribution.

#### Scanning Electron Microscopy (SEM)

Particle morphology was viewed using a Quanta 200 F field emission scanning electron microscope (FEI UK Ltd, England) operated at 10 kV in low vacuum mode and using a working distance of 10 mm. Powder samples were transferred onto glass coverslips placed onto adhesive carbon tabs (Agar Scientific, England) and mounted onto aluminium pin stubs (Agar Scientific Ltd, England). Samples were sputter coated with gold for 2 min to achieve a thickness of approximately 15–20 nm using a K550X sputter coater (Emitech, Quorum Technologies Limited, England).

#### Powder X-ray Diffraction (PXRD)

Powder X-ray diffraction (PXRD) was conducted using a Bruker D8 Advance X-ray diffractometer system (Bruker AXS Ltd, USA). X-rays were generated by a copper (Cu) source operated at a 40 kV tension and 40 mA current. Powder samples were mounted onto a zero background sample holder and scanned from 2θ = 4–30° for SX samples and 2θ = 4–35° for FP samples, with a step size of 0.039° and count time of 0.5 s per step.

#### Differential Scanning Calorimetry (DSC)

Differential scanning calorimetry (DSC) thermographs were generated using a Q Series differential scanning calorimeter (Q20 – 5023 for SX samples, Q200 – 1934 for FP samples, both TA Instruments, UK). 1 mg of sample was accurately weighed into an aluminium DSC pan (TA Instruments, UK) and hermetically sealed; for FP samples a pinhole was made in the lid prior to sealing. SX samples were heated to 160°C at heating rates of 0.1, 0.5, 1, 2, 5, 10, 20 and 40°C.min^−1^ and FP samples were heated to 320°C at a heating rate of 20°C.min^−1^, both under a nitrogen purge (50 mL.min^−1^). The DSC was calibrated at each heating rate using an indium standard.

#### Thermo-kinetic Analysis of SX Re-crystallization

Thermo-kinetic analysis of SX re-crystallization involved constructing α-heating rate curves, where α is the fraction of SX-II re-crystallized from the melt [32]. *α* was calculated using Equation 1, where *Δ*H_f_^*β* exp^ is the enthalpy of fusion of the SX-II polymorph at a given heating rate, obtained from integration of the melting endotherm, and *Δ*H_f_^*β*0.1^ is the enthalpy of fusion at the lowest heating rate employed (*i.e.* 0.1°C.min^−1^) where complete conversion of SX-I to SX-II was assumed.

1 $$ \alpha =\frac{\varDelta {H}_f^{\beta exp}}{\varDelta {H}_f^{\beta 0.1}} $$

The data were fitted to an Avrami-Erofe’ev-type equation to determine the kinetic parameters k and n, representing the integrated rate constant for the re-crystallization of SX-II and the Avrami exponent of the model respectively [32], where *β* is the heating rate.

2 $$ \alpha =1- ex{p}^{-{\left( k{\beta}^{-1}\right)}^n} $$

#### Inverse Gas Chromatography (IGC)

Surface energy analysis and specific surface area determination were conducted using an IGC Surface Energy Analyser (SEA; Surface Measurement Systems Ltd, UK). Approximately 200 mg of sample was packed into individual pre-silanised standard IGC glass column (300 mm in length, with an internal diameter of 3 mm). Each column was pre-conditioned *in situ* for 2 h at 30°C and 0%RH using helium carrier gas. Non-polar probes (n-alkanes; n-nonane, n-octane, n-heptane and n-hexane) and polar probes (ethyl acetate and dichloromethane) were injected at a range of surface coverages at 30°C using helium carrier gas at 10 sccm. Methane gas was used for dead volume corrections.

The surface coverage was calculated using the Brunauer-Emmett-Teller specific surface area (SSA_BET_) of each sample based on the n-octane adsorption isotherm data. This enabled calculation of the monolayer capacity (n_m_, the number of moles of solute adsorbed for monolayer coverage) using Equation 3, where a_m_ is the cross sectional area of a solute molecule and N_A_ is Avagadro’s number. The surface coverage (n/n_m_) at each injection concentration could then be calculated from the amount adsorbed (n) obtained from integration of the net retention volume (V_N_) *versus* the equilibrium partial pressure (P) of each injection [33].

3 $$ SS{A}_{BET}={a}_m.{N}_A.{n}_m $$

The total surface energy (*γ* ^T^) was determined from summation of the dispersive and specific surface energy components. The dispersive surface energy (*γ* ^D^) was determined by measuring the V_N_ of a series of n-alkane probes. The Dorris and Gray method was employed in this work [34], whereby a plot of RTln (V_N_) *versus* the carbon number of the n-alkanes was generated for each surface coverage. The slope of the linear regression represents the dispersive surface energy, and subsequently enables the surface energy distribution to be obtained.

Specific surface energy (*γ* ^SP^) can be determined by applying the van Oss approach [35] and the Della Volpe scale [37]. *γ* ^SP^ was calculated as the geometric mean of an acid (Lewis acceptor), *g*_*s*_^+^, and a base (Lewis donor), ${g}_{\overline{s}}$, which were determined from the injection of two monopolar probes of opposite polarities; dichloromethane (*γ* ^*y*^124.58 mJ. m^− 2^) and ethyl acetate (*γ* ^−^475.67 mJ. m^− 2^). In order to do so, specific free energy of desorption (*Δ*G_*SP*_) of the monopolar probes were first obtained using the polarisation approach [36], where a plot of RTln (V_*N*_) *versus* the molar deformation polarisation of the probes (P_*D*_) was generated. The vertical distance between each polar probe data point and the straight line of the n-alkane data was equal to the *ΔG*_*SP*_ of each polar probe [36].

Surface energy distribution profiles were generated by plotting the total, dispersive and specific surface energies at the range of surface coverages employed. This allowed the surface energy heterogeneity of the powders to be assessed.

### Powder Dispersibility

#### Dry Dispersion Laser Diffraction

A recently developed laser diffraction data analysis technique was undertaken to assess the de-agglomeration characteristics of the powder [31]. Particle size measurements were made using a Sympatec HELOS/RODOS (Sympatec GmbH, Clausthal-Zellerfeld, Germany) employing the rotary feeder and R3 lens (0.9-175 μm). Powder was hand-filled into the rotating table and the latter operated at ’20%’ rotation prior to the powder being drawn up into the dispersing line *via* a protruding aspiration tube. Particle size measurements were triggered to start when the optical concentration (C_opt_) exceeded 1.1% and ceased when the C_opt_ fell below 1% for 5 s (or 60 s real time). The timebase was 100 ms and a forced stability of ‘4’ was applied. The primary pressure (PP) was increased in the range 0.2 to 5.0 Bar. Particle size distributions (D_v10_, D_v50_, D_v90_ and VMD) were calculated using Fraunhofer theory and analyzed in WINDOX 4.0 software. Particle size measurements for a complete titration curve were made on a single day. Particle size-primary pressure profiles were constructed in order to determine the critical primary pressure (CPP, the dispersing pressure required for 100% de-agglomeration) and DA_50_ (the dispersing pressure required for 50% de-agglomeration) as described previously [31]. The CPP provided an indication of the cohesive strength of the powder and the DA_50_ is a measure of how readily powder dispersion occurs.

#### Next Generation Impactor Analysis

Cascade impaction analysis was undertaken using the NGI (*n* = 3–4) connected to a vacuum pump. Impactor plates were coated with 10 mL of coating solution (11% w/v polypropylene glycol in hexane) per plate to minimise particle bounce and re-entrainment. The NGI was assembled with 15 mL mobile phase in the pre-separator and attached to a vacuum pump. Capsules were hand filled with 10 ± 1 mg of drug powder and loaded into a Monodose device (Miat SpA, Italy). The flow rate through the NGI was adjusted to 60 L.min^−1^ ± 5% and 1 capsule was actuated for 4 s per NGI run. The device, capsules, throat, pre-separator and stages were rinsed with mobile phase and sonicated to ensure complete dissolution of deposited powder prior to analysis by HPLC. The stages were rinsed with 10 mL (stages 1–5) and 5 mL (stages 6–8) of mobile phase (with the aid of sonication) prior to analysis by HPLC.

The following parameters were calculated.

- *Recovered dose (RD)* – the total recovered dose (TRD; *i.e.* drug deposition in the device, capsules, throat, pre-separator and impactor stages) expressed as a percentage of the total actuated dose (*i.e.* powder mass weighed into the capsule)
- *Emission (ED)* – the recovered dose excluding drug retention in the device and capsules, expressed as a percentage of the TRD
- *Fine particle fraction (FPF)* – the percentage of the RD and ED with a particle size < 5.0 μm, determined from a log normal-probability plot of the cumulative mass percent of drug deposited on the impactor stages *versus* the stage cut sizes
- *Fine Particle dose (FPD) –* the mass of deposited drug with a particle size < 5.0 μm, calculated by converting the FPF into a mass using the TRD
- *Mass median aerodynamic diameter (MMAD) and geometric standard deviation (GSD) –* the median aerodynamic diameter and geometric standard deviation of the drug obtained from interpolation of the log normal-probability plot

#### High Performance Liquid Chromatography (HPLC) Analysis

Quantification of SX and FP recovery was effected using a validated HPLC method [38]. A Waters Alliance HT 2795 separations module, a Waters 2996 photodiode array detector and Waters column heater were used. The column was a Phenomenex Luna C_18_ column (150 × 4.60 mm, 3 μm) maintained at 40°C. The mobile phase consisted of 0.6% w/v ammonium acetate in ultrapure water (type 1+ graded with resistivity 18 MΩ-cm from a PureLab Ultra system, Elga LabWater, UK) and methanol in the ratio 25:75 v/v and was filtered prior to use (0.45 μm nylon filter). The mobile phase flow rate was 1 mL.min^−1^, the injection volume was 20 μL and the run time was 6 min. The detection wavelength was 228 nm. Samples were maintained at 10 ± 1°C in the autosampler chamber during analysis and peak integration was conducted using Empower Pro software (Empower 2 software, Build 2154, Waters Corporation, USA). Quantification was using pooled mixed standard calibration curves (*n* = 5) in the range 1–25 μg.mL^−1^ (R^2^; SX = 0.999, FP = 0.998) and 40–400 μg.mL^−1^ (R^2^; SX = 0.998, FP = 0.997). The limit of detection (LOD) and limit of quantification (LOQ) were 0.2 μg.mL^−1^ and 0.7 μg.mL^−1^, and 4.0 μg.mL^−1^ and 13.5 μg.mL^−1^ for the lower and upper concentration ranges of SX, respectively. The LOD and LOQ were 0.3 μg.mL^−1^ and 0.8 μg.mL^−1^, and 5.1 μg.mL^−1^ and 16.9 μg.mL^−1^ for the lower and upper concentration ranges of FP, respectively.

## RESULTS

Powder deposits were recovered from the NGI pre-separator and stages 1–7 for SX and stages 1–6 for FP when the samples were fractionated according to their aerodynamic particle size. The largest proportion of the powder deposited in the pre-separator, but within the impactor the maximum deposition occurred on stages 4 and 5 for SX, and stages 3 and 4 for FP. Therefore, these fractions were selected for full characterisation.

### Particle Size and Morphology

The PSD of the samples was measured using liquid dispersion laser diffraction. SX depositing in the pre-separator and stages 1–4 had comparable particle sizes to each other and to the unfractionated material, despite the aerodynamic cut-off sizes between the stages being different (Table I). Whereas previously the geometric particle size of powder fractions recovered from stages 1–6 of the NGI following fractionation were sequentially smaller [26], it was therefore postulated that in the current study SX powder depositing in the pre-separator and stages 1–4 remained agglomerated. Therefore agglomerates with an equivalent aerodynamic size to the stage in question deposited rather than discrete particles. SX depositing on stages 5, 6 and 7 showed a gradual shift towards a smaller particle size suggesting the deposition of individual particles may have occurred. A similar trend was observed for FP; the liquid dispersed size of pre-separator and stage 1–4 powder deposits were comparable with each other and the unfractionated material. There was a slight shift towards smaller particle sizes for the powder depositing on stages 3 and 4 that was not seen with SX, and the FP powder depositing on stages 5 and 6 had a smaller geometric size representing fully dispersed powder particles (Table I). The particle size of re-crystallized SX (D_v50_ = 4.93 ± 0.37 *μ*m) and FP (D_v50_ = 3.62 ± 1.43 *μ*m) particles was larger than their respective unfractionated powders; however, the D_v50_ was smaller than 5.0 *μ*m, the required particle size for deposition in the airways.

**Table I:** The geometric equivalent volume particle size distribution (D_v10_, D_v50_, D_v90_) of unfractionated, crystallised, and aerodynamic size-fractionated salmeterol xinafoate (SX) and fluticasone propionate (FP) sized using liquid dispersion laser diffraction (mean ± S.D., *n* = 4 and 6, respectively) and the aerodynamic cut sizes across the Next Generation Impactor when operated at 60 L.min^−1^

Sample	Aerodynamic Diameter (μm)		Salmeterol xinafoate			Fluticasone propionate		
	Minimum Size	Mid-Point Size	D_v10_ (μm)	D_v50_ (μm)	D_v90_ (μm)	D_v10_ (μm)	D_v50_ (μm)	D_v90_ (μm)
Unfractionated	n.a.	n.a.	0.62 ± 0.00	1.42 ± 0.08	3.78 ± 0.23	1.04 ± 0.40	2.94 ± 1.22	6.10 ± 2.65
Crystallised	n.a.	n.a.	0.72 ± 0.01	4.93 ± 0.37	17.4 ± 1.07	1.09 ± 0.42	3.62 ± 1.43	10.8 ± 4.67
Pre-separator	12.8	n.a.	0.64 ± 0.00	1.51 ± 0.05	3.65 ± 0.23	0.97 ± 0.01	2.39 ± 0.09	4.83 ± 0.49
Stage 1	8.06	10.2	0.65 ± 0.01	1.51 ± 0.06	3.32 ± 0.10	0.99 ± 0.03	2.41 ± 0.13	5.07 ± 0.58
Stage 2	4.46	6.00	0.65 ± 0.00	1.54 ± 0.06	3.54 ± 0.12	1.00 ± 0.03	2.48 ± 0.22	5.03 ± 0.66
Stage 3	2.82	3.55	0.66 ± 0.01	1.72 ± 0.11	3.86 ± 0.29	0.95 ± 0.02	2.14 ± 0.18	3.93 ± 0.54
Stage 4	1.66	2.16	0.64 ± 0.01	1.51 ± 0.06	3.51 ± 0.18	0.88 ± 0.01	1.91 ± 0.09	3.56 ± 0.35
Stage 5	0.94	1.25	0.61 ± 0.00	1.12 ± 0.04	2.78 ± 0.23	0.71 ± 0.02	1.59 ± 0.12	3.28 ± 0.29
Stage 6	0.55	0.72	0.59 ± 0.03	0.93 ± 0.02	2.10 ± 0.42	0.65 ± 0.02	1.46 ± 0.23	3.50 ± 0.61
Stage 7	0.34	0.43	0.55 ± 0.01	0.87 ± 0.05	1.92 ± 0.33	-	-	-

n.a. = not applicable

**Fig. 1:**
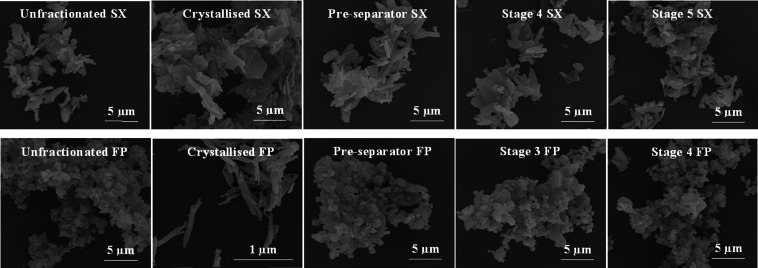
Scanning electron micrographs of unfractionated, crystallized, and aerodynamically size-fractionated salmeterol xinafoate (SX) and fluticasone propionate (FP) agglomerates at × 10500 magnification.

Particle and agglomerate morphology was unchanged between the fractionated and unfractionated powders (Fig. 1). This indicates that the fractionation process did not obviously alter particle shape, and that fractionated powders were agglomerated. The re-crystallized particles exhibited a different morphology; SX particles were more plate-like and FP particles more needle-like following re-crystallization (Fig. 1).

### Crystallinity

**Fig. 2:**
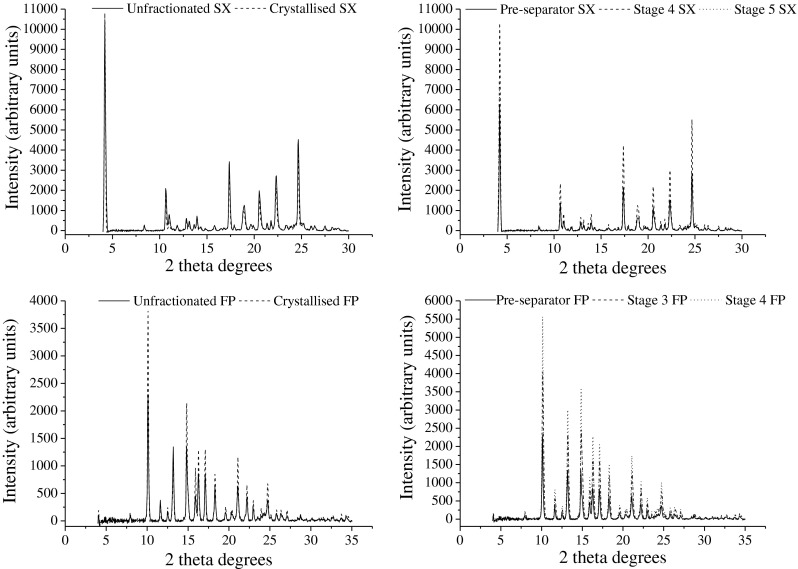
Powder x-ray diffractograms of unfractionated and crystallized salmeterol xinafoate (SX) and fluticasone propionate (FP) particles (*n* = 1 displayed).

The PXRD diffractograms of the unfractionated, crystallized and fractionated powders are shown in Fig. 2. The positions of the peaks were the same between the different samples of each powder demonstrating no change in polymorph between the unfractionated, re-crystallized and aerodynamic size-fractionated particles. DSC traces for SX samples were typical for this drug, displaying an initial endotherm due to melting of the SX-I polymorph, an exotherm due to the conversion of SX-I to the SX-II polymorph, and a second endotherm due to melting of SX-II. Increasing the heating rate revealed differences in the solid state disorder between the samples. A much higher heating rate was required to suppress melting of the SX-II polymorph for fractionated particles compared to unfractionated and crystallized particles, suggesting greater crystal damage. When modelled to a modified Avrami-Erofe’ev equation, the kinetic parameters *k* and *n*, representing the integrated rate constant for the re-crystallization of SX-II and the Avrami exponent of the model, respectively, were determined (Table II). The Avrami exponent was close to 2 for each sample, which is expected for SX particles which have a platelet shape and therefore predominantly grow in two directions [39]. The *k* values revealed a different susceptibility to re-crystallization between the samples; stage 4 and 5 particles had comparable k values whereas pre-separator particles had a much higher k value, indicating a higher degree of crystalline disorder in these particles. DSC traces for FP samples were typical for this drug undergoing melting followed by degradation [40, 41]. There was no change in the melting point between FP samples (data not shown). Although a higher background and halo effect was observable in PXRD diffractograms for stage 4 FP (Fig. 2), this could not offer conclusive evidence of altered crystallinity of FP samples due to the potential interference of orientation effects with powders in this fine size range.

**Table II:** The degree of re-crystallisation (k) and Avrami component (n) of pre-separator, stage 4 and stage 5 salmeterol xinafoate (SX) samples (mean ± SE, *n* = 3)

Sample	Degree of Re-crystallisation (*k*, °C^−1^.min)	Avrami Component (*n*)
Pre-separator SX	8.12 ± 0.26	2.08 ± 0.17
Stage 4 SX	6.29 ± 0.28	2.18 ± 0.25
Stage 5 SX	6.66 ± 0.27	1.96 ± 0.18

### Surface Energy Distribution

**Fig. 3:**
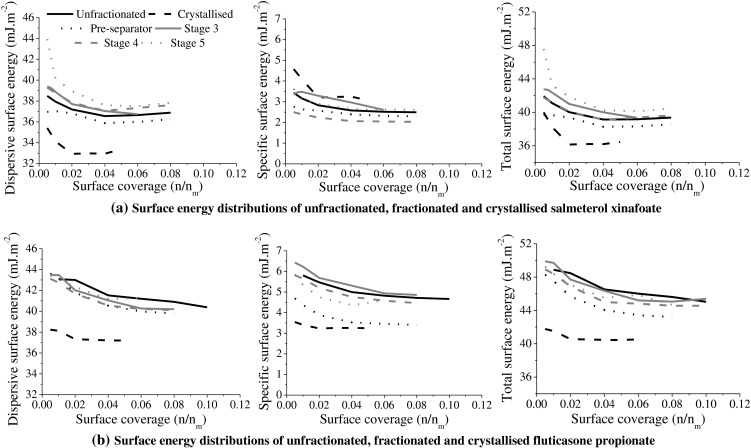
The dispersive, specific and total surface energy distributions of unfractionated, crystallized, and aerodynamic size-fractionated (**a**) salmeterol xinafoate (SX) and (**b**) fluticasone propionate (FP) particles (NB the same legend is used for each graph).

The surface energy of the powders was characterised in terms of the dispersive, polar and total surface energy (Fig. 3). The results showed a gradual decrease in the surface energy value as a function of surface coverage, and the extent of the decline can be inferred as a marker of surface energetic inhomogeneity. For all the powders, the dispersive surface energy formed the major component of the total surface energy. Both SX and FP re-crystallized particles had lower total surface energy than the micronized (*i.e.* unfractionated) particles. For FP crystals, the specific surface energy (relating to polar interactions) and dispersive surface energy (relating to van der Waals interactions) were both lower than the micronized (*i.e.* unfractionated) FP particles. For SX crystals, there was an increase in the specific surface energy and a reduction in the dispersive surface energy compared to the micronized (*i.e.* unfractionated) SX particles.

In terms of the total surface energy, unfractionated and Stage 4 SX powders had similar surface energy, Stage 3 SX had slightly higher and more heterogeneous surface energy than unfractionated SX, whereas Stage 5 particles had the highest and most heterogeneous surface energies of all the fractionated SX samples. Pre-separator SX had the lowest surface energy of the fractionated material. For FP a different trend was observed in that all the fractionated samples had lower surface energy than the unfractionated FP powder however the differences were small. Again, pre-separator FP had the lowest surface energy of the fractionated samples.

### Powder re-dispersal and aerosolization

The dispersibility of the powders was assessed using a laser diffraction data analysis technique in which it is possible to determine the ease of de-agglomeration (DA_50_) and cohesive tendencies (CPP) of the powders (Table III). Following data normalization and linearization as previously described [31], the linearity was found to be good (R^2^ = 0.892–0.998 for SX, R^2^–0.906–0.968 for FP). For SX, in all instances (except for Stage 5 particles), the DA_50_ was lower compared to unfractionated SX suggesting an improvement in dispersibility; pre-separator and Stage 4 particles showed the largest improvement. For Stage 5 particles the DA_50_ was comparable to that of the unfractionated SX indicating an equally poor dispersibility. FP samples showed a different behaviour. Pre-separator FP displayed a much larger DA_50_ value compared to the unfractionated FP indicating worsened dispersibility. The remaining powders showed an improved ease of dispersion with a rank order of Stage 4 > crystallized > Stage 3 FP in terms of a reducing DA_50_.

**Table III:** The primary pressure for 50% de-agglomeration (DA_50_) and critical primary pressure (CPP) of unfractionated, crystallised and aerodynamic size-fractionated salmeterol xinafoate (SX) and fluticasone propionate (FP) assessed by dry dispersion laser diffraction

	DA_50_ (Bar)	CPP (Bar)
Salmeterol xinafoate		
Micronised	1.45	3.50
Crystallised	1.21	n.a
Pre-separator	0.54	1.20
Stage 4	0.51	3.50
Stage 5*	1.40	3.00
Fluticasone propionate		
Micronised	1.72	n.a.
Crystallised	1.05	3.50
Pre-separator	2.36	n.a
Stage 3	0.85	n.a.
Stage 4	1.50	n.a.

*Data modelled in the PP range 0.3 – 5.0 Bar; for all other samples PPs were in the range 0.2 – 5.0 Bar; n.a. = not possible to assign a CPP.

It was not always possible to deduce a measure of powder cohesive strength according to the criteria previously described [31]. This could arise from high heterogeneity in particle properties, incomplete de-agglomeration (even at the highest dispersing pressure employed) or conversely particle fracture/attrition occurring at high dispersing pressures. It was therefore not possible to compare the CPP of the FP samples. For SX, only pre-separator particles showed a reduction in cohesive strength compared to the unfractionated powder (Table III).

Cascade impactor analysis also revealed differences in the aerosolization of the samples (Table IV). For SX, the RDs were in the range 72 ± 2.6% to 87 ± 1.3%. Despite a larger particle size, re-crystallized SX exhibited a much higher emission (91 ± 2.1%; one-way ANOVA with post-hoc Tukey’s test, *p* < 0.05) than unfractionated particles (54 ± 5.2%), potentially due to reduced adhesion to device/capsule walls and lower cohesivity facilitating flow and entrainment. However, an increase in deposition in the throat and pre-separator for these larger particles resulted in no overall change in the FPF (*p* > 0.05) compared to the unfractionated powder. Pre-separator SX also exhibited higher emission (by approximately 10%) compared to unfractionated SX powder (p > 0.05). The FPF of pre-separator and unfractionated SX was unchanged (*p* > 0.05) such that although pre-separator particles were entrained more readily, the de-agglomeration efficiency was lower than for the unfractionated material. The emission of unfractionated, Stage 4 and Stage 5 SX samples did not differ significantly, and there was no change in FPF between Stage 4 and the unfractionated powder (*p* > 0.05). The FPF of Stage 5 SX, however, was reduced compared to unfractionated SX (*p* < 0.05) suggesting poorer de-agglomeration efficiency of the emitted mass.

**Table IV:** The emission (% of the total recovered dose, RD), fine particle fraction (FPF < 5.0 μm, % RD and % emitted dose, ED), fine particle dose (FPD < 5.0 μm), mass median aerodynamic diameter (MMAD) and geometric standard deviation (GSD) of unfractionated, crystallised and aerodynamic size-fractionated salmeterol xinafoate (SX) and fluticasone propionate (FP) assessed by Next Generation Impactor analysis at 60 L.min^−1^ (mean ± SD, *n* = 3–4)

	Emission (% RD)	Fine Particle Fraction (% RD)	Fine Particle Fraction (% ED)	Fine Particle Dose (mg)	Mass Median Aerodynamic Diameter (μm)	Geometric Standard Deviation
Salmeterol xinafoate						
Unfractionated	54.4 ± 5.2	33.2 ± 2.2	61.2 ± 2.5	2.7 ± 0.2	2.7 ± 0.1	1.8 ± 0.0
Crystallised	91.0 ± 2.1	27.7 ± 1.6	30.4 ± 2.4	2.4 ± 0.2	3.5 ± 0.2	2.1 ± 0.0
Pre-separator	64.1 ± 6.9	38.4 ± 4.6	59.8 ± 2.4	3.0 ± 0.4	2.6 ± 0.1	1.8 ± 0.0
Stage 4	47.3 ± 3.9	33.8 ± 5.1	71.3 ± 6.0	2.7 ± 0.5	2.5 ± 0.1	1.8 ± 0.0
Stage 5	44.8 ± 2.6	20.6 ± 1.9	45.9 ± 2.9	1.4 ± 0.0	2.7 ± 0.1	2.0 ± 0.0
Fluticasone propionate						
Unfractionated	62.2 ± 7.6	18.7 ± 0.4	30.4 ± 4.0	1.5 ± 0.1	4.1 ± 0.3	2.0 ± 0.1
Crystallised	60.4 ± 2.9	25.2 ± 3.3	40.8 ± 6.6	2.2 ± 0.3	4.1 ± 0.2	2.0 ± 0.1
Pre-separator	68.2 ± 4.1	22.3 ± 0.8	32.9 ± 2.8	1.8 ± 0.0	4.1 ± 0.1	2.1 ± 0.2
Stage 3	59.6 ± 3.4	31.6 ± 1.7	53.1 ± 0.8	2.5 ± 0.1	3.7 ± 0.1	1.9 ± 0.0
Stage 4	58.7 ± 3.6	30.5 ± 0.6	52.1 ± 2.9	2.5 ± 0.1	3.5 ± 0.1	1.9 ± 0.0

FP samples exhibited different trends in aerosolization compared to SX samples (Table IV). The RD of FP ranged from 78 ± 1.7% to 90 ± 2.0%. The emission of re-crystallized FP particles was unchanged compared to unfractionated FP (*p* > 0.05) however the FPF increased (*p* < 0.05). The emission of pre-separator, Stage 3 and Stage 4 FP was unchanged compared to the unfractionated powder (*p* > 0.05). The stage fractions showed an increase in their FPF compared to unfractionated FP (*p* < 0.05), indicating an improvement in de-agglomeration efficiency.

## DISCUSSION

Particle production for delivery to the lungs commonly involves crystallization followed by a comminution step such as micronization in order to achieve an appropriate particle size for deposition in the lungs. The highly energetic micronization process can result in poor control over the physicochemical properties of the particles with the potential for both intra- and inter-batch variability [15]. A consequence of this could be the generation of populations of particles which display different physicochemical and aerosolization behaviour to the bulk of the powder. For example, the introduction of amorphous regions onto crystalline drug surfaces can increase surface energy and adhesive/cohesive forces [3, 42] such that particles may be more difficult to fluidize and/or de-agglomerate; there may even be a proportion of particles that are so tightly agglomerated that they do not disperse during delivery. A recently developed aerodynamic technique which enables powders to be separated based on their aerodynamic particle size [26] has therefore provided the possibility to collect and study discrete powder fractions for their properties. The fractionation methodology was extended in the current study to examine the physicochemical properties of aerodynamically differentiated material, and therefore allow the effect of intra-batch variability in particle properties present within bulk powders to be determined.

During DPI delivery, powder agglomerates must disperse within an airstream in order to attain a particle size which is able to deposit in the lungs. For both SX and FP, the particle size (expressed as the D_v50_) of the pre-separator and stage fractions (stage 1–3 for FP and stage 1–4 for SX) following dispersion in liquid media were comparable to the unfractionated powders (Table I) suggesting that the particles remained agglomerated during fractionation and when depositing in the NGI. The stage cut sizes therefore represented the aerodynamic size of the agglomerates rather than single particles of equivalent size, shape or density. Categorical evidence for the deposition of agglomerates within the cut-off size ranges of impactor stages has not previously been reported, although it has been postulated [26, 43]. The particle size of stage 4–6 deposits for FP, and stage 5–7 deposits for SX, were smaller than the unfractionated particle size and similar to the stage aerodynamic size range, suggesting that these particles may have dispersed more fully prior to deposition.

To provide a crystalline comparator, re-crystallized particles were produced by Amphiphilic Crystallization [27] such that the particles would not possess any process-induced changes to their physicochemical properties. Although the re-crystallized particles were larger than the micronized (*i.e.* unfractionated) material, the D_v50_ was below 5.0 μm and therefore likely to be within the aerodynamic size range suitable for pulmonary delivery. Achieving such a particle size is one of the major challenges of controlled crystallization methods, as small molecules tend to form relatively large crystals (10–100 μm) and the crystal size distribution is dependent on the crystallization conditions [1, 27, 28]. As the primary aim was to obtain particles which had not been subjected to secondary processing to function as control material in terms of bulk and surface crystallinity, rather than to obtain the smallest possible crystal size, the crystallization method was not further optimized in this study.

Although there was no change in particle morphology, (by visual assessment, Fig. 1) between the fractionated and unfractionated powders, there were differences in other physicochemical properties. PXRD analysis did not identify a change in polymorphic form between any of the samples, however, differences in crystallinity (subsequently referred to as ‘bulk disorder’) were observed by DSC for SX samples. The fractionated samples were found to have higher bulk disorder than the unfractionated powder. The stage fractions had comparable bulk disorder to each other, which in turn was lower than the pre-separator fraction. Higher disorder may arise from particle damage during powder processing such as the use of micronization [44]. Levels of disorder generated are dependent on the amount of energy imparted during size reduction [16, 45] and milling time [46, 47]. It is understandable that a powder should show distribution in the degree of disorder within a single batch of powder. The crystal damage observed for fractionated SX samples may not have been detected in the unfractionated material due to these more severely damaged particles being present in a much smaller proportion in the bulk material. Nevertheless, small quantities of these particles may be important in terms of controlling the dispersion of the bulk into the constituent particles upon aerosolization. The most damaged particles were found to have the lowest propensity for de-agglomeration during fractionation, as particles in the pre-separator sample formed larger agglomerates (>12.8 μm) compared to those of the Stage 4 and 5 samples (0.55–1.66 μm).

Despite having the highest bulk disorder, pre-separator SX particles had the lowest and least heterogeneous total surface energy when compared to the unfractionated and stage fractionated samples (Fig. 3). Stage 4 SX possessed near-identical surface energy to unfractionated SX, with Stage 5 SX displaying the highest and most heterogeneous surface energy. The smallest Stage 5 particles are likely to have been subjected to the highest degree of particle attrition during comminution and potentially demonstrate the greatest particle damage [16, 45–47], manifesting as high bulk (represented by the k value) and surface (represented by the surface energy) disorder. Higher dispersive surface energies have been reported with increasing milling energy and grinding pressures, with an inverse relationship with the geometric particle size (*i.e.* with reducing size) resulting for drug particles following micronization [48, 49]. These findings were attributed to greater crystal disruption due to greater forces during micronization, and the formation of new highly energetic interaction sites and/or the exposure of more energetic crystal faces [48, 49]. The balance of the dispersive and polar interactions also differed between the fractionated samples, for example, Stage 4 SX had higher and lower dispersive and specific surface energies, respectively, than the unfractionated powder, whereas for pre-separator SX the surface energies were both lower than the unfractionated powder. This suggests that size reduction of the particles may have occurred by different particle size reduction mechanisms (*e.g.* brittle fracture or attrition) and/or at different cleavage planes, resulting in altered exposure of functional groups at the crystal surface between the samples [49].

In contrast to SX, the differences in surface energy between FP samples were overall less marked. Pre-separator particles had the lowest total surface energy, whereas for the unfractionated and stage fractions the surface energy could be considered broadly comparable. Once more, differences in the relative contributions of the dispersive and specific surface energies were observed, indicating that the fractionated materials consisted of particles with differing electron donating and accepting functional groups at the exposed crystal surface [49]. In addition, differences in the degree of surface energy heterogeneity were observed for FP fractions.

Each powder fraction was found to display distinct aerosolization behaviours when the fraction was considered as a bulk powder and re-aerosolized. A powder composed of pre-separator material would be likely to have a structure that comprised of large agglomerates (>12.8 μm) exhibiting good flow [50]. Low inter-agglomerate cohesive forces are likely due to large agglomerate sizes, however, poor dispersal during fractionation would suggest that intra-agglomerate cohesive forces between the individual component particles was high. Both SX and FP pre-separator samples were emitted in greater amounts and displayed a higher FPF than the respective unfractionated powders (Table IV), although the differences were not significant (*p* > 0.05). Low inter-agglomerate cohesive forces would assist the efficient entrainment of powder agglomerates into the airstream during aerosolization. High inter-particulate forces between individual particles may have restricted the efficiency of the fractionation, but during re-aerosolization of the smaller powder mass into the NGI then the powder structure would be altered compared to the unfractionated starting material. The efficiency of de-agglomeration therefore depends on the balance between high inter-particulate cohesive forces, and the higher aerodynamic drag forces and kinetic energy experienced by large agglomerates (compared to smaller agglomerates), which in the latter case may increase de-agglomeration efficiency through a greater number of collisions/impaction within the device [49].

Changes in the intrinsic dispersibility of the pre-separator fractions assessed by dry dispersion laser diffraction, however, differed between the drugs and to the aerosolization behaviour seen in the NGI. Whereas the SX pre-separator sample showed an improvement in the ease of de-agglomeration (DA_50_), for FP the DA_50_ was worsened compared to the respective unfractionated powders. The CPP also revealed a difference in the cohesive strength of the fractionated SX samples; there were no/small differences between the CPP of the stage fractions, but the CPP of pre-separator SX was lower than that of the unfractionated material, concurring with the lowest total surface energy of this sample compared to the stage fractions and unfractionated material. Whereas cascade impactor analysis considers powder entrainability (*i.e.* emission) and de-agglomeration efficiency (*i.e.* FPF) as separate parameters, the ease of dispersibility (*i.e.* DA_50_) as determined using the laser diffraction technique incorporates the flow, entrainment and de-agglomeration of the powder, independent of the method of delivery (*i.e.* device and flow rate), into a single, powerful parameter for assessing powder dispersion, along with enabling a measure of bulk powder cohesive strength (CPP). A further advantage is that measurements take into account every type of interactive force present, as well as powder structure and history [31]. The structural characteristics of a powder play an important role in dictating de-agglomeration patterns [52, 53] and are influenced by the physicochemical properties of the individual particles. The work of cohesion (dependent on factors such as the surface energy distribution of the particles), packing fraction (*i.e.* volume of particles/volume of agglomerate, and related to the structure of agglomerates resulting from the magnitude and distribution of interactive forces) and the size of the individual particles, dictate powder structure, and can result in a lack of structure homogeneity across the powder bed [52, 54]. It is therefore necessary to consider both the fundamental dispersibility of the powder and the powder delivery system, including the device, formulation and flow rate, when assessing powders for their de-agglomeration efficiency, and developing delivery systems for optimized fine particle delivery to the lungs.

The aerosol performance of the stage fractions differed between the drugs. Stage 4 SX, despite having an improved DA_50_, showed no change in emission or FPF compared to unfractionated SX. Stage 5 powder when re-aerosolized, however, displayed a comparable, poor, DA_50_ and a reduced FPF (*p* < 0.05). When combined with the absence of change in the emission, the high DA_50_ and reduced FPF indicated a poorer de-agglomeration efficiency of Stage 5 SX. In this instance, a combination of a small particle size (Table I), high bulk disorder (Table II) and high surface disorder (Fig. 3) resulted in a reduction in aerosol performance, where it is likely that strong cohesive forces (CPP = 3.0 Bar, Table III) between the particles led to the formation of stable agglomerates that were difficult to disperse [51]. Conversely, stage fractionated FP (*i.e.* both Stage 3 and 4 particles) showed an improvement in the DA_50_ and an increase in the FPF (*p* < 0.05), which alongside no change in emission indicated an improvement in de-agglomeration efficiency compared to unfractionated material. This improvement could not be attributed to changes in crystallanity or (total) surface energy between the unfractionated and stage fractionated FP particles, and highlights the complexity in the factors that influence the de-agglomeration process. It was also not possible to deduce a CPP for the unfractionated or fractionated FP samples, despite a change in the DA_50_. The particle size-dispersing pressure profiles were characteristic of micronized powders and flattened as the dispersing pressure increased [31], however, examination of this region of the curve indicated that the particle size continued to reduce up to 5.0 Bar. The magnitude of the reduction was small in comparison to the early region of the curve (*e.g.* Stage 3 FP had a D_v50_ of 12.29 μm, 2.75 μm and 1.80 μm at 0.2, 1.5 and 5.0 Bar, respectively). It is likely that a combination of particle heterogeneity and particle fracture/attrition (as a result of high dispersing pressures) resulted in the lack of a plateau region according to the criteria previously described [31].

The lower surface energy and larger size of the re-crystallized control particles [30, 55] resulted in improved dispersibility in terms of the DA_50_ for both drugs, entrainability for SX and de-agglomeration efficiency for FP compared to the unfractionated powders. The FPFs of precipitated drug crystals have been shown to be higher than jet-milled particles for both disodium chromoglycate [14] and salbutamol sulphate [56], and equivalent for SX [30]. FP crystals have demonstrated improved [3], or similar FPFs [30] depending on the crystallization conditions employed. In part, the improvements observed in this study are likely to arise from the emission of the high energy micronization step, however, other factors such as altered surface chemistry, and changes to particle shape, particularly for FP, also need to be considered and require further investigation.

The aerodynamic size fractions isolated in this study were found to have their own distinct physicochemical properties, which although in isolation could not fully describe the different aerosolization behaviours of the fractions, highlighted the consequences of intra-powder variability on aerosol performance. It is generally accepted that a micronized powder will show a distribution in its particle size and surface energy, and we have further demonstrated that it is possible to characterise the sub-populations of a powder in these terms and determine the effects on aerosolization. It is therefore appropriate to consider powder properties as distributions when seeking to investigate and optimise de-agglomeration behaviour rather than using ‘average’ values [25] which may not provide an accurate representation of true powder character. In particular, the ability to identify fractions of powders with optimal dispersibiltiy may offer the opportunity to prepare carrier-based blends of micronized powders with greater homogeneity in powder blending and aerosolization performances.

## CONCLUSION

This study has demonstrated that SX and FP powders fractionated into distinct aerodynamic size classes comprised of sub-populations of the bulk micronized powder. These sub-populations consisted of individual particles agglomerated to varying extents and displaying distinct physicochemical properties of crystallinity and surface energy. It is important to consider powders as collections of particle populations with their own distinct properties; i.e. there is a need to consider powder properties as distributions. By gaining better control over the generation and properties of sub-populations of particles, it may be possible to optimize the efficient delivery of drug particles to the lungs.
